# *BRAF* gene mutations in synchronous papillary thyroid carcinoma and Langerhans cell histiocytosis co-existing in the thyroid gland: a case report and literature review

**DOI:** 10.1186/s12885-019-5372-3

**Published:** 2019-02-22

**Authors:** Mohammad A. Al Hamad, Hassan M. Albisher, Weam R. Al Saeed, Ahmed T. Almumtin, Fatimah M. Allabbad, Mohammed A. Shawarby

**Affiliations:** 1 Department of Pathology, College of Medicine, Imam Abdulrahman Bin Faisal University, Dammam, Saudi Arabia; 2 Department of Surgery, College of Medicine, Imam Abdulrahman Bin Faisal University, Dammam, Saudi Arabia; 3 Department of Pathology and Laboratory Medicine, King Fahad Hospital of the University, PO Box 2208, Khobar, 31952-2208 Saudi Arabia

**Keywords:** BRAF gene mutations, Papillary thyroid carcinoma, Langerhans cell histiocytosis

## Abstract

**Background:**

Langerhans cell histiocytosis (LCH) is a rare clonal disease, characterized by hyperproliferation of Langerhans cells. It may rarely involve the thyroid gland. Its association with papillary thyroid carcinoma (PTC) is extremely rare; with only few case reports available in the English literature. *BRAF* mutations are implicated in the development of papillary thyroid carcinoma, and have also been identified in Langerhans cell histiocytosis.

**Case presentation:**

Here we present a rare case of a 36-year-old Indonesian female patient with dysphagia associated with neck mass which was complicated by skin sinus formation. The diagnosis of PTC was rendered on fine needle aspiration (FNA). Debulking thyroidectomy revealed co-existeence of PTC and LCH. On subsequent molecular testing, *BRAF V600E* and *V600K* mutations were detected in tissues macrodissected from both lesions, respectively. To the best of our knowledge, this case is the first case to report two different *BRAF* mutations in tissues of a Langerhans cell histiocytosis and a papillary thyroid carcinoma co-existing in the thyroid gland. The patient received chemotherapy of etoposide combined with prednisone. At the most recent follow-up, the patient is in a stable clinical condition.

**Conclusions:**

The coexistence of a PTC with LCH harboring *BRAF* mutation may suggest etiologic relation between the two conditions that involves the *BRAF* gene. Clinically, it may suggest an aggressive, locally advanced thyroid cancer, an impression that may reflect on the selected surgical management, chemotherapy and *BRAF* mutation-targeting therapy to these patients.

## Background

Thyroid cancer is the commonest malignancy of the endocrine system [1]. Most primary thyroid cancers originate from the epithelial cells that line the thyroid follicles. There are four main types of thyroid carcinoma, namely, papillary, follicular, medullary and anaplastic. Langerhans cell histiocytosis (eosinophilic granuloma) is a rare disease, characterized by a wide range of clinical presentations including isolated lesions, multiple lesions or multi organ disseminated disease. The disease is characterized by hyperproliferation of Langerhans cells that share similarities with antigen-presenting Langerhans cells of mucosal sites, thymus, lymph nodes and skin [2]. LCH does not arise from epidermal Langerhans cells, but from misguided myeloid dendritic cell precursors [3]. The debate over whether LCH is a neoplasm or a reactive inflammatory disorder has been discussed over many years. Evidence of clonality in LCH was reported in 1994 and, thereby, it is currently considered as a neoplastic process [2]. LCH may rarely involve the thyroid gland. Its association with PTC is also extremely rare, with only few case reports available in the literature [1–8]. Table 1 represents the cases of synchronous papillary thyroid carcinoma and Langerhans cell histiocytosis co-existing in the thyroid gland [1, 2, 7–16]. Herein, we report a case of co-existing PTC and LCH in the thyroid gland with detected *BRAF V600E* and *V600K* mutations respectively.

**Table 1:** Reported cases of synchronous co-existing of PTC and LCH in the thyroid gland

Author/Year	Sex/Age	PTC&LCH In Thyroid	Side	LCH in other organs	Treatment	BRAF mutations	Follow up (months)
Goldstein N, 1991 [9]	F/31	Yes	Left	Bone, Pituitary gland, Lung, Skin, Vagina	Surgery, Prednisone, Vincristine, Methotrexate, Chlorambucil	NR	DF (6)
Saiz E, 2000 [10]	M/43	Yes	Left	No	Surgery	NR	DF(24)
Foulet-Roge A, 2002 [11]	F/42	Yes	Left	No	Surgery	NR	DF (14)
Burnett A, 2008 [12]	M/3	LCH in Right, PTC in Left	Bilateral	Lung	Surgery, Prednisone, Mercaptopurine, Methotrexate	NR	NR
Jamaati HR, 2009 [13]	M/24	Yes	Bilateral	Lung	Surgery, Etoposide, Dexamethasone	NR	DF(NR)
Vergez S, 2010 [1]	M/29	Yes	Bilateral	Bone, Pituitary gland, Lung, Skin	Corticosteroids, Vinblastine, Cladribine, Imatinib	NR	DRD(36)
Chung DH, 2012 [2]	F/53	Yes	Right	NR	Surgery	NR	NR
Ceyran AB, 2014 [14]	M/37	Yes	Bilateral	NR	Surgery	NR	Died due to Cardiac arrest
Gordon M S, 2016 [16]	F/22	Yes	Bilateral	Labia Vulva	Surgery, Prednisone	V600E in PTC	NR
Alzahrani R, 2016 [7]	F/27	Yes	Bilateral	No	Surgery, CT Prednisone	NR	NR
Wu X, 2017 [8]	M/40	Yes	Right	Lung, Liver	Surgery, CT	NR	DF(24)
Jaimanti Bakshi JK,2018 [15]	M/31	Yes	Right	No	Surgery, Vinblastine and Etoposide	NR	NR
Current case	F/37	Yes	Bilateral	No	Surgery, Prednisone with Etoposide	V600E in PTC,V600K in LCH	DF (12)

*F* female, *M* male, *CT* chemotherapy, *NR* not recorded, *DF* disease free, *DRD* death related disease

## Case presentation

A 36-year-old woman was referred to the endocrine surgeon with a neck mass that gradually enlarged over a year. This mass was associated with dysphagia and complicated by skin sinus formation. There was no prior or family history of thyroid disease. Laboratory tests showed no evidence of thyroid hyper or hypo function.

**Fig. 1:**
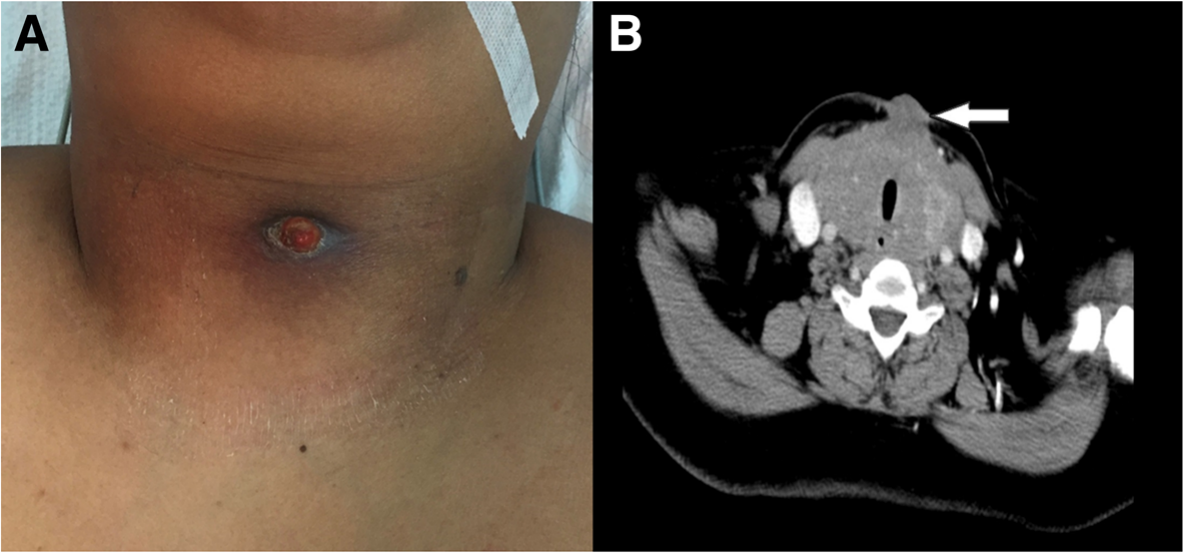
Anterior midline neck sinus secondary to infiltrating thyroid cancer (**a**); CT scan transverse view: diffuse enlargement of thyroid gland infiltrating the skin with heterogeneous density (**b**)

Physical examination revealed a central neck skin sinus with a serosanguinous discharge (Fig. 1a). The thyroid gland was non-tender, diffusely enlarged, and extending bilaterally and retrosternally, mainly on the left side, with no cervical lymphadenopathy detected. Computerized tomography (CT) scan demonstrated a diffuse enlargement of the thyroid gland with skin infiltration, heterogeneous density and macrocalcification. The thyroid enlargement caused tracheal and esophageal compression and displaced neck vasculature laterally (Fig. 1b). Brain, chest, abdomen and pelvis CTs did not show any metastasis.

FNA from the right thyroid lobe was interpreted as PTC. The decision was to go for total thyroidectomy with central neck dissection. Intraoperatively, the thyroid gland was extremely adherent to the surrounding structures with infiltration of the skin, strap muscles and both carotid sheaths, and extensively adherent to the esophagus and trachea. Consequently, only debulking thyroidectomy (isthmectomy) was performed along with sinus track excision and sampling of the central lymph node compartment.

**Fig. 2:**
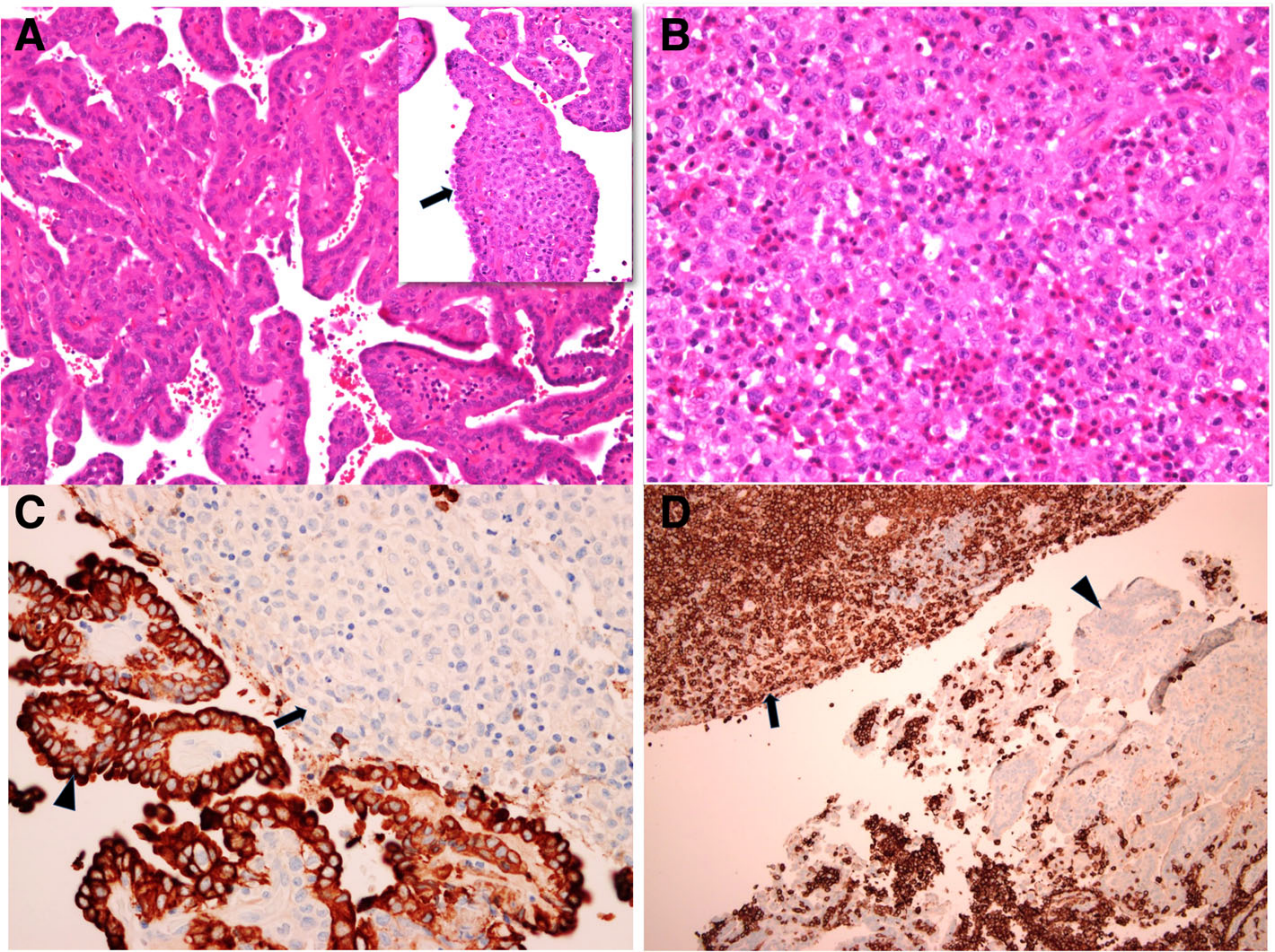
Coexisting LCH and PTC. Focally extended to cores of PTC (inset, arrow), H&E × 400 (**a**); Sheets of LCH cells replacing thyroid parenchyma, H&E × 200 (**b**); PTC cells staining positively for panCK (arrow head); LCH cells are not stained (arrow), IHC × 400 (**c**); LCH cells staining positively for CD1a (arrow); PTC cells are not stained (arrow head), IHC × 100 (**d**)

The thyroid specimen was received fragmented and consisted of multiple pieces of firm brown-tan tissue, measuring 5.5 × 5 × 0.4 cm in aggregate. Also received was an ellipse of skin and a “central” lymph node, measuring 5x1x0.7 cm and 1.5 × 0.8x4cm, respectively. Microscopic examination of sections from the thyroid gland revealed a classical PTC (Fig. 2a). In addition, there were cells with eosinophilic cytoplasm and mild to moderately pleomorphic nuclei, some showing irregular contours or grooves, admixed with inflammatory cells including numerous eosinophils. These cells were seen in extensive sheets replacing 60–70% of the resected thyroid tissue as well as focally within the neoplastic papillary cores (Fig. 2b). Similar cell sheets were seen in sections of the skin and lymph node. Immunohistochemically, the papillary carcinoma stained positively for pan cytokeratin (CK) (Fig. 2c) and cytokeratin 19 (CK19) (data not shown), while the infiltrating mononuclear cells showed diffuse, strong reactivity for CD1a (Fig. 2d), CD43, S100 protein and were negative for myeloperoxidase, LCA and HMB45 immunostians (data not shown). The Ki67 proliferation index in these cells was around 60% (data not shown). The final diagnosis was synchronous PTC and LCH involving thyroid, overlying skin and cervical lymph node.

**Fig. 3:**
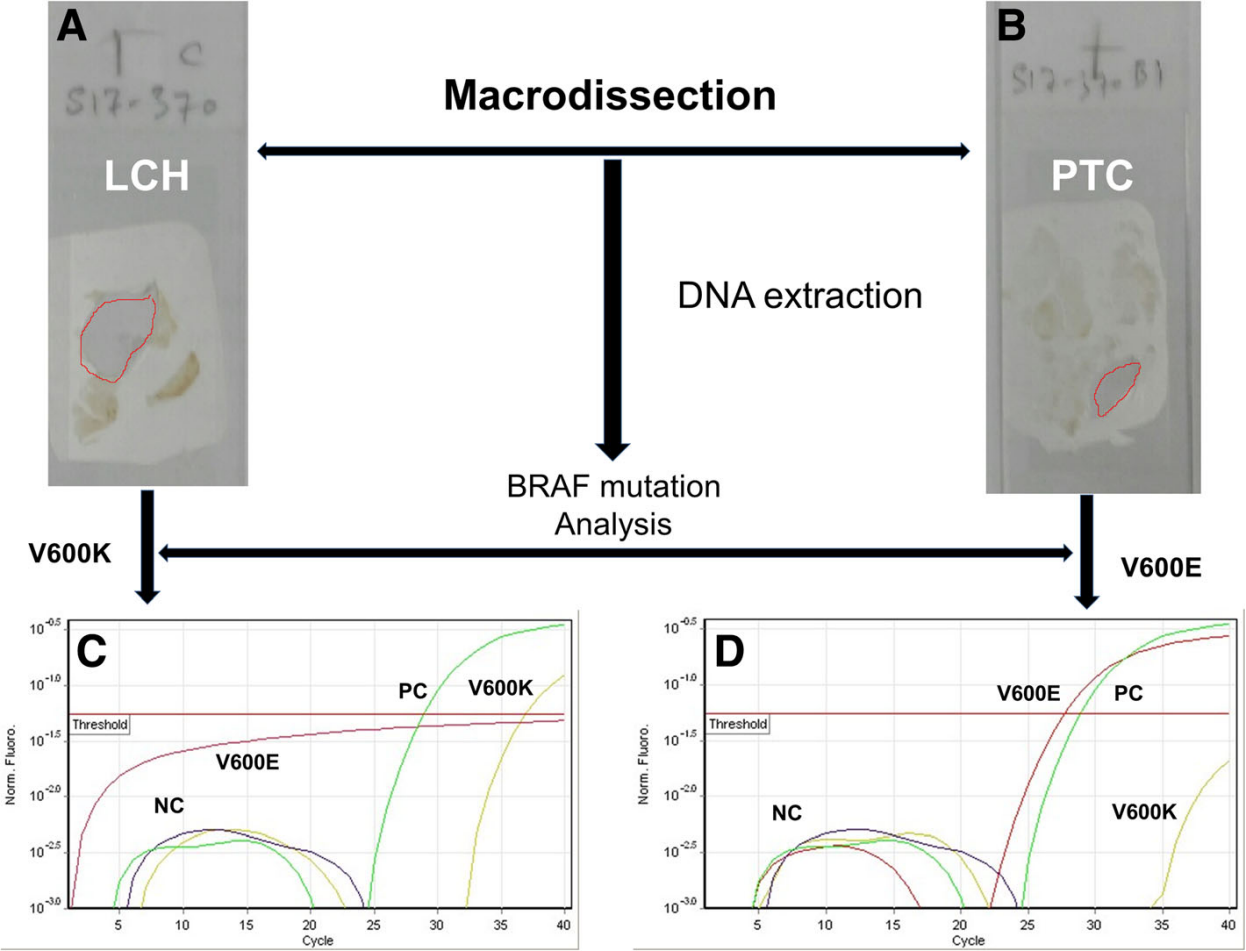
*BRAF* gene mutations of LCH and PTC co-existing in the thyroid gland. Macrodissected LCH cells (marked red) from the thyroid gland (**a**); macrodissected PTC cells (marked red) from the thyroid gland (**b**); V600k BRAF mutation detected in the DNA extracted from LCH cells (**c**); V600E BRAF mutation detected in the DNA extracted from PTC cells (**d**); PC: positive control; NC: negative control

Paraffin blocks including both the PTC and the LCH were manually macrodissected. DNA was automatically extracted and purified by QIAcube (Qiagen) from the two lesions separately using QIAamp DNA FFPE, while the concentration and purity of extracted DNA was assessed by Epoch-Bioteck. *BRAF* mutations were investigating using Easy BRAF kit (Diatech) that detects BRAF codon 600 mutations by real-time PCR (Rotor-Gene). DNA was assessed by analysis of the reaction controls (water, BRAF positive control) and analysis of BRAF control mix. The two DNA samples extracted from the two different lesions passed the assessment of DNA, and then were analyzed to search for mutations. Mutational analysis of *BRAF* for the DNA extracted from PTC and LHC revealed *V600E* and *V600K* mutations, respectively (Fig. 3).

The postoperative course was uneventful. The patient received palliative chemotherapy, at her home country, that consisted of Etoposide with Prednisone. After twelve months of follow-up, the patient is in a stable clinical condition.

## Discussion and conclusions

LCH has a broad spectrum of clinical presentations and may rarely involve the thyroid gland. There is probably some association between LCH and PTC, as few cases of LCH co-existing with PTC have been previously described in the English literature (Table 1).

Activating *BRAF* mutations is an important event in the development of PTC [17, 18]. *BRAF V600E* mutation has been reported in about 40–60% of patients with PTC [19]. A somatic activating *BRAF V600E* mutation has also been identified in approximately 60% of LCH, strongly supporting the neoplastic rather than inflammatory nature of LCH [20]. However, no sufficient information is available in the literature, regarding the *BRAF* mutation status of cases of co-existing LCH and PTC [1]. It has been speculated that the *BRAF V600E* mutation in LCH could lead to PTC through creating a microenvironment that is appropriate for neoplastic transformation [4]. To the best of our knowledge, our case is the first report of *BRAF* mutations in tissues of a LCH and a PTC co-existing in the thyroid gland, suggesting an aggressive disease and etiologic relation between the two conditions that involves the *BRAF* gene. Since the patient decided to start the treatment at home country, there was limitation in the patient follow-up. Our case is the second case reported to harbor *BRAF V600K* mutation in LCH [21]. The PTC was not iatrogenically induced as the patient did not receive any prior radiotherapy [22], while LCH patients showed improvement after chemotherapy [8].

The co-existence of LCH with an otherwise classical PTC may lead to a clinical impression of an aggressive, locally advanced type of thyroid cancer. This may affect the selection of surgical management. This was our experience in the present case in which only debulking thyroidectomy was attempted to relieve the compressive symptoms, as total thyroidectomy was not possible due to widespread local extension and the involvement of vital surrounding structures.

The clinical behavior of LCH is extremely variable. Some cases are limited in extent and follow an indolent course, whereas others exhibit multi organ involvement, frequent recurrences, and are refractory to conventional therapy. The presence *BRAF V600E* mutation was shown to be associated with high-risk features and poor response to chemotherapy in children with LCH [23]. Clinically and histologically, our case exhibited features suggestive of an aggressive behavior, namely, an extensively infiltrative pattern, lymph node involvement and a high Ki67 proliferation index.

In conclusion, co-existing PTC and LCH in the thyroid gland is extremely rare. The demonstration of *BRAF* mutations in tissues obtained from both lesions suggests an etiologic relation between the two conditions and opens the door for further studies to investigate a possible etiologic connection between LCH and PTC.

The co-existence of BRAF mutated-LCH with an otherwise classical PTC may lead to a clinical impression of an aggressive, locally advanced type of thyroid cancer. This may have an impact on the surgical procedure selection, as well as post-surgical treatment.

## Abbreviations

CT: Computerized tomography
FNA: Fine needle aspiration
LCH: Langerhans cell histiocytosis
PTC: Papillary thyroid carcinoma
